# Cytokine secretion responsiveness of lymphomonocytes following cortisol cell exposure: Sex differences

**DOI:** 10.1371/journal.pone.0200924

**Published:** 2018-07-26

**Authors:** Eleonora Da Pozzo, Chiara Giacomelli, Chiara Cavallini, Claudia Martini

**Affiliations:** Department of Pharmacy, University of Pisa, Pisa, Italy; Univerzitet u Beogradu, SERBIA

## Abstract

The stress hormone cortisol has been recognized as a coordinator of immune response. However, its different ability to modulate the release of inflammatory mediators in males and females has not been clarified yet. Indeed, the dissection of cortisol specific actions may be difficult due to the complex hormonal and physio-pathological individual status. Herein, the release of inflammatory mediators following increasing cortisol concentrations was investigated in an *in vitro* model of primary human male and female lymphomonocytes. The use of a defined cellular model to assess sex differences in inflammatory cytokine secretion could be useful to exclude the effects of divergent and fluctuating sex hormone levels occurring *in vivo*. Herein, the cells were challenged with cortisol concentrations resembling the plasma levels achieving in physiological and stressful conditions. The production of cytokines and other molecules involved in inflammatory process was determined. In basal conditions, male cells presented higher levels of some pro-inflammatory molecules (NF-kB and IDO-1 mRNAs, IL-6 and kynurenine) than female cells. Following cortisol exposure, the levels of the pro-inflammatory cytokines, IL-6 and IL-8, were increased in male cells. Conversely, in female cells IL-6 release was unchanged and IL-8 levels were decreased. Anti-inflammatory cytokines, IL-4 and IL-10, did not change in male cells and increased in female cells. Interestingly, kynurenine levels were higher in female cells than in male cells following cortisol stimulus. These results highlighted that cortisol differently affects male and female lymphomonocytes, shifting the cytokine release in favour of a pro-inflammatory pattern in male cells and an anti-inflammatory secretion profile in female cells, opening the way to study the influences of other stressful factors involved in the neurohumoral changes occurring in the response to stress conditions.

## Introduction

Sex has been reported as one of the several variables interfering with the occurrence and severity of stress-related disorders. Men are more predisposed to infectious diseases, hypertension, drug abuse and aggressive behaviour. On the other hand, conditions such as autoimmune diseases, chronic pain, anxiety disorders and depression are prevalent among women [1–4]. The differences in stress reactivity have been proposed as an important risk factor for such sex-specific diseases [5–6]. Differences based on sex in response to stress have been occasionally reported in healthy subjects too. These responses are related both to the type and time of stress exposure, and to the pathophysiological and hormonal status of the individuals [7–8]. In such a complex background, the papers are numerous but the data are sometimes controversial. Therefore, it is important to provide data obtained from well-controlled experimental settings to shed light on the molecular mechanisms at the heart of stress responses. A cellular model could be also useful to control the divergent and fluctuating sex hormone levels occurring *in vivo* that could affect the stress responses. A defined *in vitro* model, previously used to assess the alteration of immune response secretion following stress-mimicking conditions, is the human cell culture of peripheral blood mononuclear cells (PBMCs) exposed to cortisol. Cortisol is the major stress hormone and it has been recognized as a modulator of inflammatory responses. In literature, PBMCs have been exposed to stress-mimicking settings and alterations of cytokine secretion have been observed [9–10]. In light of this, the purpose of the study was the assessment of male and female PBMCs responses following cortisol treatment. In particular, the cells were exposed to a low cortisol concentration, simulating the physiological cortisol level, and to a higher cortisol concentration similar to that occurring in plasma following acute stress. Through immune-enzymatic assays and real-time reverse transcription polymerase chain reaction (real-time RT-PCR) techniques, interleukins (the pro-inflammatory interleukins, IL-6 and IL-8, and the anti-inflammatory interleukins, IL-4 and IL-10) and other molecules involved in inflammatory processes (NF-kB transcription factor, indoleamine 2,3-dioxygenase 1 -IDO-1- and kynurenine -Kyn-) were investigated. The results showed significant differences between male and female PBMCs responses under cortisol exposure.

## Materials and methods

### Materials

The frozen human peripheral blood mononuclear cells were obtained from Zen-Bio Inc. (Research Triangle Park, NC, USA). As the European Pharmacopoeia’s guideline recommendation for a cellular test investigating cytokine output (the monocyte-activation test (2.6.30) published in the European Pharmacopoeia Supplement 9.2), each PBMC pools (male and female PBMCs) was prepared from four healthy donors, in order to reduce the effects of donor variability. Healthy donors were matched for race (Caucasian) and age (31±3). Each cryovial contained similar content of lymphocytes and monocytes, as reported in the furnished data sheets (lymphocytes = 89.35% ± 1.91; monocytes = 10,65% ± 1.91).

Cell culture media and foetal bovine serum were obtained from Cambrex Bio Science Walkersville, Inc. (Walkersville, MD). Cortisol (HC, hydrocortisone ≥98% (HPLC), powder) was obtained from SIGMA Italia (Milan, Italy). The Human IL-6 ELISA Kit, Human IL-10 ELISA Kit and Human IL-8 ELISA Kit were obtained from Thermo Fisher Scientific Inc. (Rockford, USA). The Human IL-4 ELISA Kit was obtained from Cloud Clone Corp (Katy, USA). The Human KYN ELISA Kit was obtained from Cusabio Biotech Co (Baltimore Avenue, College Park, USA). All other reagents were from standard commercial sources.

### Cell culture, treatments and preparations

The human male and female PBMCs were defrosted in RPMI 1640 medium supplemented with 10% foetal bovine serum, 2 mM of L-glutamine, 100 U/ml of penicillin, and 200 microg/ml of streptomycin, distributed in 24-well multiplates (5 x 10^5^ cells/well) or in 10 cm-dishes (5 x 10^6^ cells) and maintained in a humidified atmosphere of 95% air/5% CO_2_ at 37°C. After 24 hours, PBMCs were treated with increasing concentrations of cortisol in the serum free cell medium. Hydrocortisone stock solutions were prepared by dissolving HC in ethanol and then diluting with pyrogen-free sterile saline solution (NaCl 0.9%) to achieve equipotent final culture concentrations of 0.2 microg/ml (physiological plasma cortisol level) and 0.4 microg/ml (plasma cortisol level in high-stress status) [11]. PBMCs were added with NaCl 0.9% (control) and treated with ethanol 0.1% or cortisol to final cortisol concentrations of 0, 0.2 and 0.4 microg/ml.

Following a 16 h incubation period, cells were collected and centrifuged for 10 min at 2000xg at 4°C. Such an incubation period was needed to achieve the optimal expression of inflammatory molecules, as indicated by previous time course experiments [12]. The supernatant was collected and stored at -80°C until assayed. Aliquots of cells were suspended in an appropriate buffer (phosphate saline buffer containing a protease inhibitor cocktail) and lysed through multiple freeze/thaw cycles. The homogenates were centrifuged for 5 min at 5000xg at 4°C, and the supernatant was collected. Protein concentrations of cell supernatants and cell lysates were estimated by the method described by Lowry et al. [13] using bovine serum albumin as a standard. Lysates were collected and stored at -80°C until assayed. Other aliquots of cells were immediately processed for the RNA isolation using the RNeasyH Mini Kit.

#### Cytokine assays

Supernatant levels of IL-6, IL-10, IL-8 and IL-4 were determined using commercial enzyme-linked immunosorbent assay (ELISA). The most highly sensitivity ELISAs were chosen to measure the concentrations of IL-6 (Thermo Fisher Scientific Inc., detection range: 10.2 to 400pg/mL, sensibility < 1 pg/mL), IL-10 (Thermo Fisher Scientific Inc., detection range: 15.4 to 600 pg/mL, sensibility < 3 pg/mL), IL-8 (Thermo Fisher Scientific Inc., detection range: 25.6 to 1000 pg/mL, sensibility < 2 pg/mL), and IL-4 (Cloud Clone Corp., detection range: 15.6–1000 pg/mL, sensibility < 5.7 pg/mL).The effect of cortisol treatment on the rate of cytokine secretions was measured.

#### Cytokine Secretion Impact (CSI)

To better visualize the cortisol effects on cytokine production, the results were analysed in an integrated fashion (multi-cytokine sensor). This approach was developed from a similar method used to discriminate the quality of specific water samples [14]. The sensors were used to calculate the Index of Cytokine Secretion Impact (CSI), using ImageJ software (National Institute of Mental Health, Bethesda, Maryland, USA). The CSI represents the ratio between the secretion of inflammatory (IL-6 and IL-8) and anti-inflammatory (IL-4 and IL-10) cytokines. Briefly, data were represented using the Radar Plot on Microsoft Excel 2010. Radar plots were generated by reporting the IL-6, IL-10, IL-4 and IL-8 mean values as a set of points (interleukin points) plotted along a set of axes radiating from a central point. The interleukin points were connected by a line. The areas under the curve (AUC) were obtained by ImageJ analyses, and the ratio pro-/anti-inflammatory effects were calculated. For a 0<CSI<1, the cytokine secretion profile of PBMC is mainly anti-inflammatory; for a CSI>1, the cytokine secretion profile is primarily inflammatory.

This analysis enabled us to dissect the sex impact on pro-inflammatory or anti-inflammatory cytokine secretion profiles during stimulation of different cortisol concentrations.

#### Relative mRNA quantification of NFkB and IDO-1 genes

The relative quantification of interleukins, NFkB and IDO-1 mRNA was performed by real-time RT-PCR as previously described [15] in PBMCs. In brief, total RNA was isolated using the RNeasy Mini Kit (Quiagen). The purity of the RNA samples was determined by measuring the absorbance at 260:280 nm. cDNA synthesis was performed with 500 ng of RNA using the iScript cDNA Synthesis Kit (Bio Rad). The primers used for the RT-PCR were designed to span intron/exon boundaries to ensure that products did not include genomic DNA (Table 1).

**Table 1:** Nucleotide sequences, product size and annealing temperature of the primers utilized in real-time RT-PCR experiments.

Gene	Primer nucleotide sequences	Product size (base pairs)	Annealing Temperature
IL-6	FOR: 5′-TCCTCGACGGCATCTCA-3′ REV: 5′-TTTTCACCAGGCAAGTCTCCT-3′	165 bp	55°C
IL-8	FOR: 5′-AAGAGAGCTCTGTCTGGACC-3′ REV: 5′-GATATTCTCTTGGCCCTTGG-3′	408 bp	56°C
IL-10	FOR: 5′-CAAGCTGAGAACCAAGACCC-3′ REV: 5′-AAGATGTCAAACTCACTCATGGC-3′	141 bp	55°C
IL-4	FOR: 5′-ACTTTGAACAGCCTCACAGAG-3′ REV: 5′-TTGGAGGCAGCAAAGATGTC-3′	74 bp	56°C
NFkB	FOR: 5′-CAGCAGATGGCCCATACCTT-3′ REV: 5′-CACCATGTCCTTGGGTCCAC-3′	287 bp	55°C
IDO-1	FOR: 5′-GATGAAGAAGTGGGCTTTGC-3′ REV: 5′-TCCAGTTTGCCAAGACACAG-3′	352 bp	55°C
beta-actin	FOR: 5′-GCACTCTTCCAGCCTTCCTTCC-3′ REV: 5′-GAGCCGCCGATCCACACG-3′	254 bp	55°C

The RT-PCR reactions consisted of 25 microL of BrilliantH II SYBRH Green premix, 1.5 microL of both the forward and reverse primers (at a 10 mM concentration), 3 microL of cDNA and 19 microL of H_2_O. All reactions were performed for 40 cycles using the following temperature profiles: 98°C for 30 seconds (initial denaturation), 55°C for 30 seconds (annealing) and 72°C for 3 seconds (extension). Beta-actin was used as the housekeeping gene. PCR specificity was determined using both a melting curve analysis and gel electrophoresis, and the data were analysed by the standard curve method. Interleukins, NFkB and IDO-1 mRNA levels for each sample were normalised against beta-actin mRNA levels, and relative expression was calculated using the Ct value.

#### Kynurenine quantification

The Human KYN ELISA Kit from Cusabio Biotech Co. was used to quantify the Kyn amount in cellular lysates, according to manufacturer’s instructions. The detection range of the kit was 0.12–500 pmol/ml, and the sensitivity was 0.12 pmol/ml. Kyn concentration was normalized to the protein content; the rationale for this correction is the fact that the lysing process caused a great variation among lysates in protein content.

#### Statistical analyses

All calculations were performed using Excel 2013 and the nonlinear multipurpose curve-fitting program GraphPad Prism that was also used for statistical analysis and graphic presentations. AUC for CSI calculation were obtained by the use of the open source Java image processing program ImageJ. All data are presented as the means ± SEM. Statistical analysis was performed by a t-test or a one-way analysis of variance (ANOVA) with the Newman-Keuls multiple comparison test for post hoc pair-wise comparisons. Two-way ANOVA analyses were also performed to determine how the responses were affected by sex and cortisol concentrations. The results were considered significant at the p<0.05 level.

## Results

### Pro-inflammatory cytokine production in cortisol treated PBMCs

Male and female PBMCs were exposed to different cortisol concentrations mimicking physiological cortisol levels. In particular, the 0.2 and 0.4 microg/ml doses were chosen to resemble the normal plasma cortisol level and the cortisol level in highly stressed persons, respectively, in accordance with previous literature [16].

**Fig 1:**
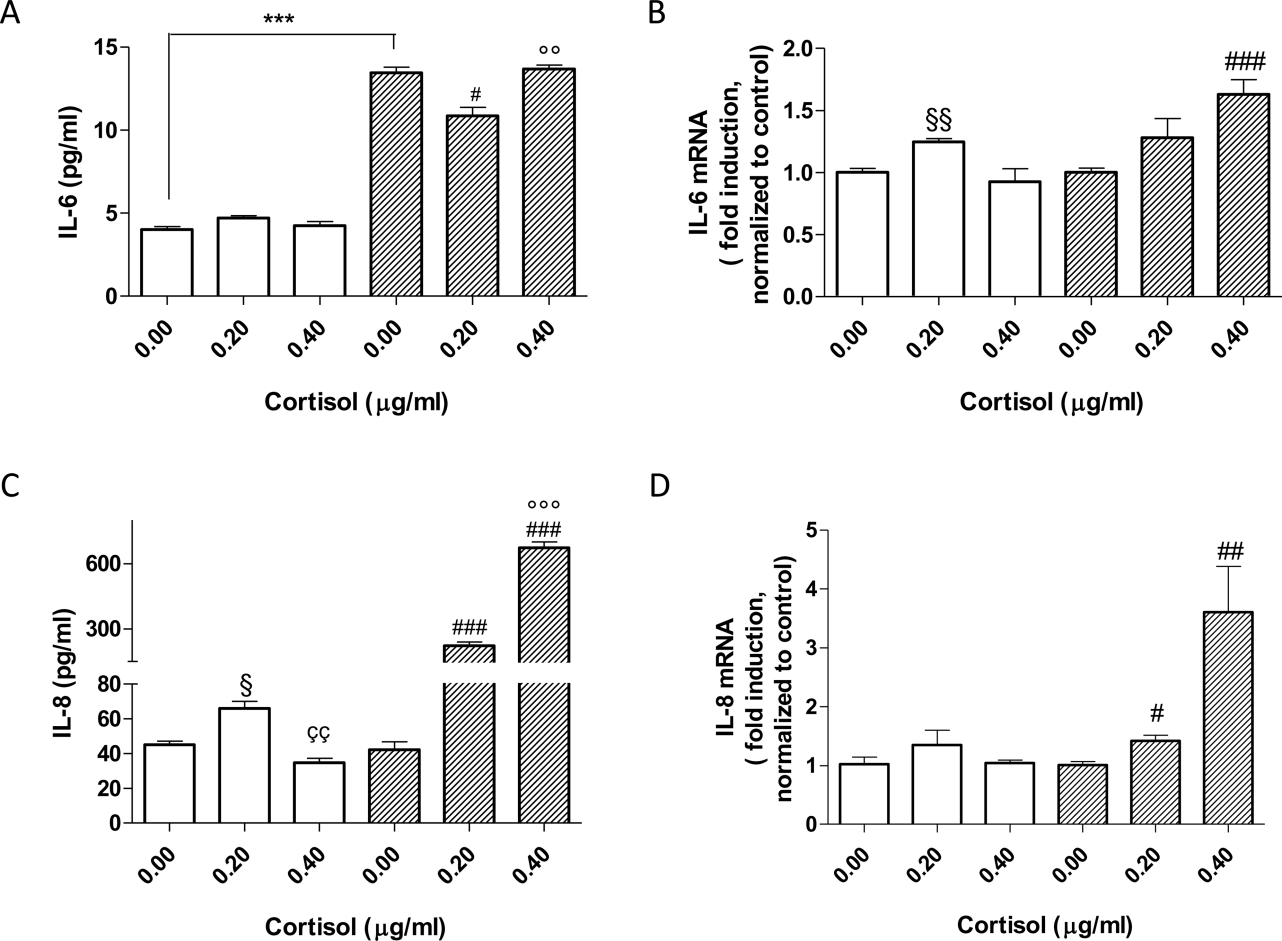
Pro-inflammatory interleukin secretion and gene expression following cortisol challenges. The white bars represent the mean ± SEM of three values performed in triplicate in female PBMCs; the striped bars represent the mean ± SEM of three values performed in triplicate in male PBMCs. *** p<0.001 vs female cells (t-test); # p<0.05 or ### p<0.001 vs not-stimulated male cells (One-way Anova, Newman-Keuls post test); § p<0.05 vs not-stimulated female cells (One-way Anova, Newman-Keuls post test); çç p<0.01 vs 0.2 microg/ml cortisol treated female cells (t-test); °° p<0.01 or °°° p<0.001 vs 0.2 microg/ml cortisol treated male cells (t-test). For IL-6, Two-way ANOVA test (sex x treatment): interaction p value = 0.0001; F = 19.50; DFn = 2. For IL-8, Two-way ANOVA test (sex x treatment): interaction p value < 0.0001; F = 392.7; DFn = 2.

The levels of pro-inflammatory cytokines were first assessed in unstimulated PBMCs evidencing that the levels of IL-6 in basal condition were significantly higher in male (striped) than in female (in white) PBMCs (Fig 1A; ***, p<0.001), whereas the IL-8 did not show sex-related differences (Fig 1B). Following physiological cortisol stimulation, the IL-6 levels were not significantly changed in female cells (in white), while in male PBMCs cortisol caused a significant immunosuppressive effect (#, p<0.05 vs not stimulated condition) (Fig 1A), despite the levels remaining significantly higher with respect to the female cells. Interestingly, following the highest cortisol treatment, the IL-6 release in female PBMCs was not affected. In contrast, such a concentration of cortisol in male cells was able to increase the IL-6 secretion with respect to physiological cortisol (°° p<0,01, Fig 1A). Two-way ANOVA tests confirmed the differences in the cortisol treatment for the IL-6 release between the two sexes; the interaction is considered extremely significant (Interaction P value = 0.0001; F = 19.50; DFn = 2).

In order to dissect the mechanism at the basis of cortisol effects, the IL-6 and IL-8 gene expression was also evaluated (Fig 1B and 1D). In particular, the IL-6 mRNA levels were comparable to the cytokine secretion in female cells. Conversely, the intracellular gene expression of IL-6 in male cells was significant increase. The data confirmed that cortisol was able to differently modulate the expression of IL-6 based on sex.

Then, the levels of the pro-inflammatory cytokine IL-8 were assessed (Fig 1C). IL-8 is a cytokine produced by mononuclear cells that is involved in cellular recruitment and activation [17], and it is released from PBMCs during the first hours of an infection [18]. As reported in Fig 1, panel c, unstimulated male and female PBMCs did not show differences in IL-8 levels. Under physiological cortisol treatment, cells of both sexes increased IL-8 secretion but with different extents; male cells produced higher levels of interleukin with respect to female cells (§, p<0.05 in female PBMCs; ###, p<0.001). Furthermore, in the presence of the highest cortisol concentration, female cells significantly decreased IL-8 secretion (çç, p<0.01), while male PBMCs further increased the IL-8 levels with respect to physiological cortisol stimulation (°°°, p<0.001). These data were completely in accordance with the ability of cortisol to modulate the IL-8 gene expression (Fig 1D).

Two-way ANOVA analyses proved that the cortisol treatments affect male and female cell responses differently; the interaction was considered extremely significant (interaction p value < 0.0001; F = 392.7; DFn = 2).

### Anti-inflammatory cytokine productions in cortisol treated PBMC

**Fig 2:**
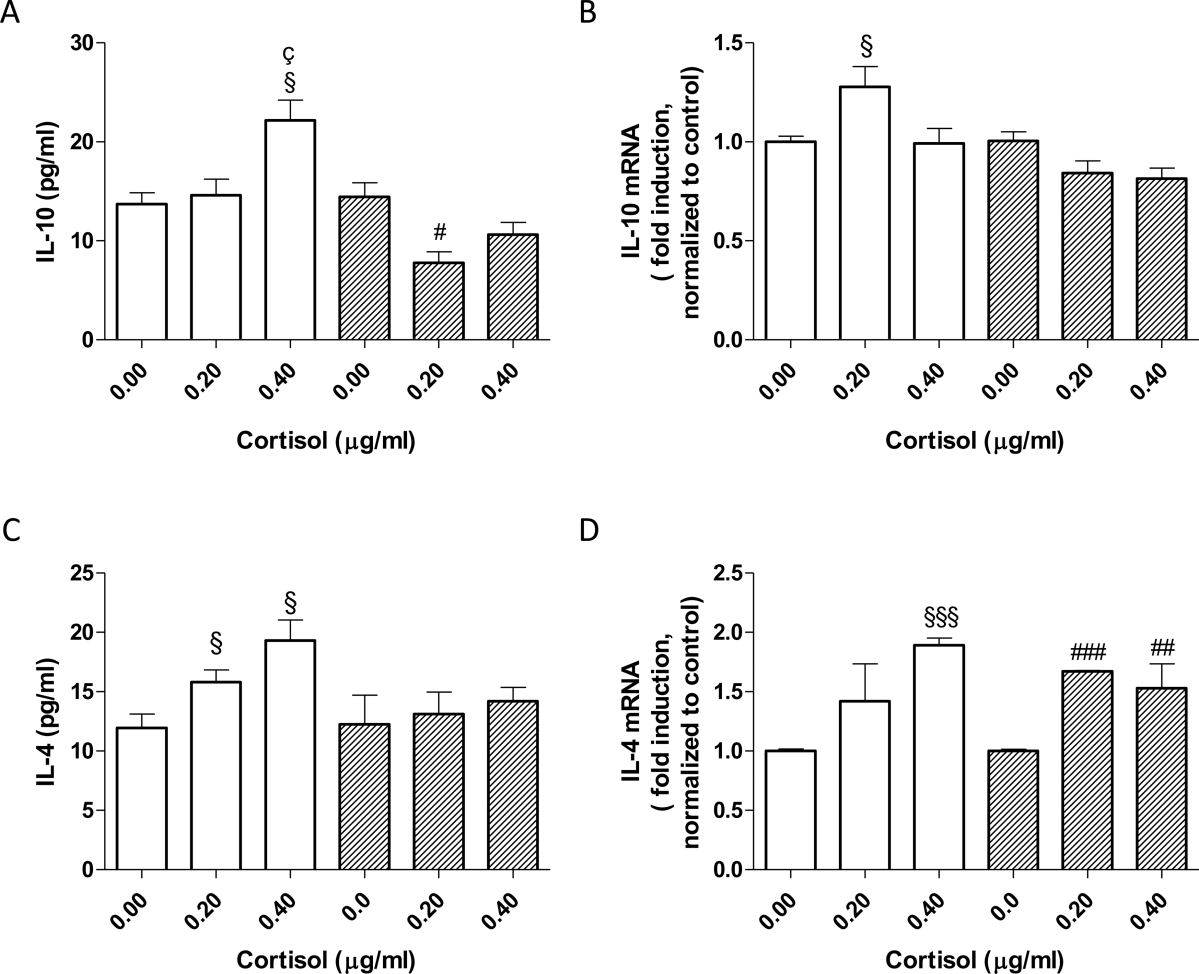
Anti-inflammatory interleukin secretion and gene expression following cortisol challenges. The white bars represent the mean ± SEM of three values performed in triplicate in female PBMCs; the striped bars represent the mean ± SEM of three values performed in triplicate in male PBMCs. § p<0.05 vs not-stimulated female cells (One-way Anova, Newman-Keuls post test); # p<0.05 vs not-stimulated male cells (One-way Anova, Newman-Keuls post test); ç p<0.05 vs 0.2 microg/ml cortisol treated female cells (t-test). For IL-10, Two-way ANOVA test (sex x treatment): interaction p value = 0.0043; F = 7.64; DFn = 2. For IL-4, Two-way ANOVA test (sex x treatment): interaction p value = 0.2402; F = 1.51; DFn = 2.

To further investigate the modulation of PBMC secretions under different cortisol concentrations, the levels of the anti-inflammatory cytokines IL-10 and IL-4 were investigated. Both the interleukins did not show sex-related differences in unstimulated cells (Fig 2).

Following physiological cortisol stimulation, the IL-10 release by female PBMCs was not significantly affected, while in male cells, cortisol demonstrated immunosuppressive effects (#, p<0.05 vs not stimulated condition) (Fig 2A). The highest cortisol concentration caused a significant IL-10 increase in female cells (°, p<0.05 vs physiological cortisol; Fig 2A) and only a slight increase in male cells. Two-way ANOVA test demonstrated that the cortisol had different effects in males and females; the interaction was considered very significant (interaction p value = 0.0043; F = 7.64; DFn = 2). In accordance with these data, cortisol was able to increase the expression of IL-10 in female cells with different extent based on cortisol concentrations (Fig 2B). Conversely, the hormone was able to slightly decrease the IL-10 production in male cells, explaining the decrease of the cytokine release.

IL-4 has anti-inflammatory properties in monocytes [19]. A physiological cortisol concentration was able to increase the IL-4 secretion in female PBMCs (§, p<0.05, Fig 2B); on the contrary, male cells did not show significant differences in the IL-4 levels (Fig 2C). The highest cortisol concentration did not significantly affect the IL-4 release pattern in both sexes with respect to the physiological response; in female cells, a significant increase was evidenced. Conversely, in male cells, there was only a slight change. These trends were in accordance with the gene expression pattern of IL-4 (Fig 2D) in both sexes. The two-way ANOVA tests indicated no sex difference for IL-4 following cortisol treatment (p = 0.2402; F = 1.51; DFn = 2).

### Determination of the pro- and anti-inflammatory cytokine balance using the multi-cytokine sensor

**Fig 3:**
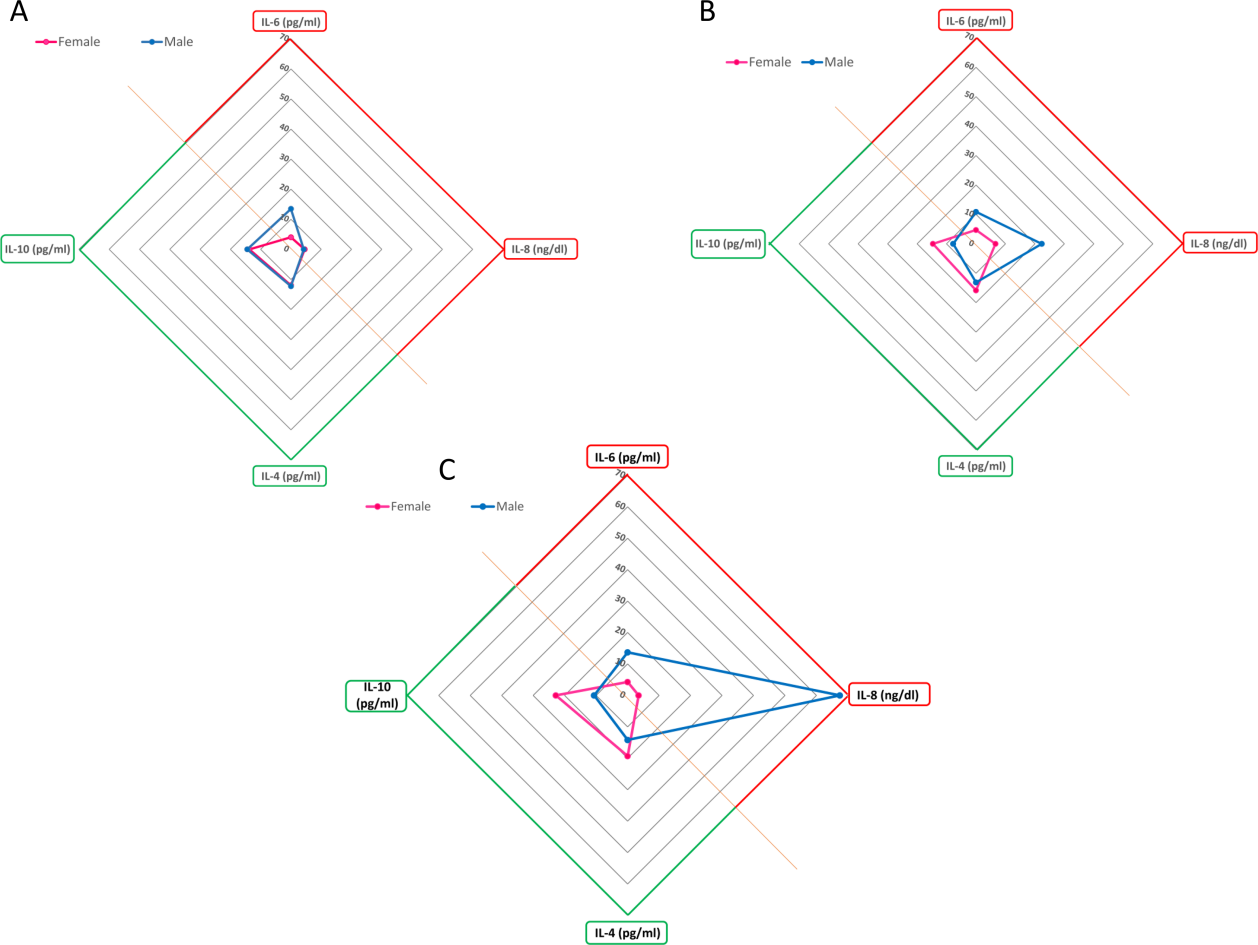
Use of multi-cytokine sensors analyse the balance between pro- and anti-inflammatory cytokine release following cortisol challenges. A) pro-/anti-inflammatory balance in non-stimulated PBMCs; B) pro-/anti-inflammatory balance in physiological cortisol-stimulated PBMCs; C) pro-/anti-inflammatory balance in high cortisol-stimulated PBMCs. Pro-inflammatory interleukins are represented in red; anti-inflammatory interleukins are represented in green. The orange line represents the border between the anti- (under) and pro- (above) inflammatory secretion areas.

The multi-cytokine sensor reported in this study allowed the investigation of the balance between inflammatory and anti-inflammatory interleukins secreted by PBMCs following cortisol treatment. The response of the multi-cytokine sensor was evaluated in the absence or presence of different cortisol concentrations, plotting the data obtained in male (blue line) and female (pink line) PBMCs (Fig 3). The results of unstimulated cells (Fig 3A), cells treated with physiological cortisol concentration (Fig 3B) and cells treated with the highest cortisol concentration (Fig 3C) were reported. It was evident that the secretion pattern of male cells had a trend in favour of pro-inflammatory cytokines in the presence of increasing cortisol concentrations. On the contrary, female cells produced a more pronounced anti-inflammatory secretion pattern in the presence of increasing cortisol concentrations.

Then, the multi-cytokine sensors were analysed using ImageJ software to calculate the CSI index. Data are reported in Table 2.

**Table 2:** Values of the CSI index.

Cell Treatment Condition	Sex	Total area	PI area	AI area	CSI
**Non-stimulated cells** **(0 microg/ml)**	Male	0.427	0.132	0.285	**0.463**
	Female	0.264	0.027	0.246	**0.110**
**Physiological cortisol** **(0.2 microg/ml)**	Male	0.597	0.410	0.194	**2.113**
	Female	0.356	0.069	0.292	**0.236**
**Acute stress cortisol** **(0.4 microg/ml)**	Male	1.634	1.390	0.274	**5.073**
	Female	0.489	0.042	0.456	**0.092**

The values are reported as the ratio between the secretion areas of pro-inflammatory (IL-6 and IL-8), PI, and anti-inflammatory (IL-4 and IL-10), AI, cytokines. For 0<CSI<1, the cytokine secretion profile of PBMC is mainly anti-inflammatory; for CSI>1, the cytokine secretion profile is principally pro-inflammatory.

As reported in Table 2, PBMCs increased the cytokine release in response to increasing concentrations of cortisol, as attested by the rise in total area values in response to cortisol doses. In particular, this increase was more evident in male cells with respect to female cells.

Furthermore, the CSI values highlighted that cortisol treatment, and in particular, the highest concentration, oppositely affected male and female cells, shifting the cytokine release in favour of a pro-inflammatory secretion profile in male cells and of an anti-inflammatory secretion profile in female lymphomonocytes. In fact, in the presence of both cortisol concentrations, the CSI index were <1 for female and >1 for male (Table 2).

### IDO-1 expression and kynurenine production in cortisol treated PBMC

The enzyme IDO-1 is constitutively expressed in blood monocytes [20], and inflammatory cytokines such as interferon, IL-1 beta and IL-6 during an immune response are able to increase its expression [21]. IDO-1 catalyses the first and rate-limiting step of tryptophan oxidation and creates an immunosuppressive microenvironment decreasing the levels of the essential amino acid tryptophan and producing immunosuppressive tryptophan metabolites such as kynurenine [22]. IDO-1 has been generally considered an important counter-regulatory enzyme, which controls disproportionate immune responses [23].

**Fig 4:**
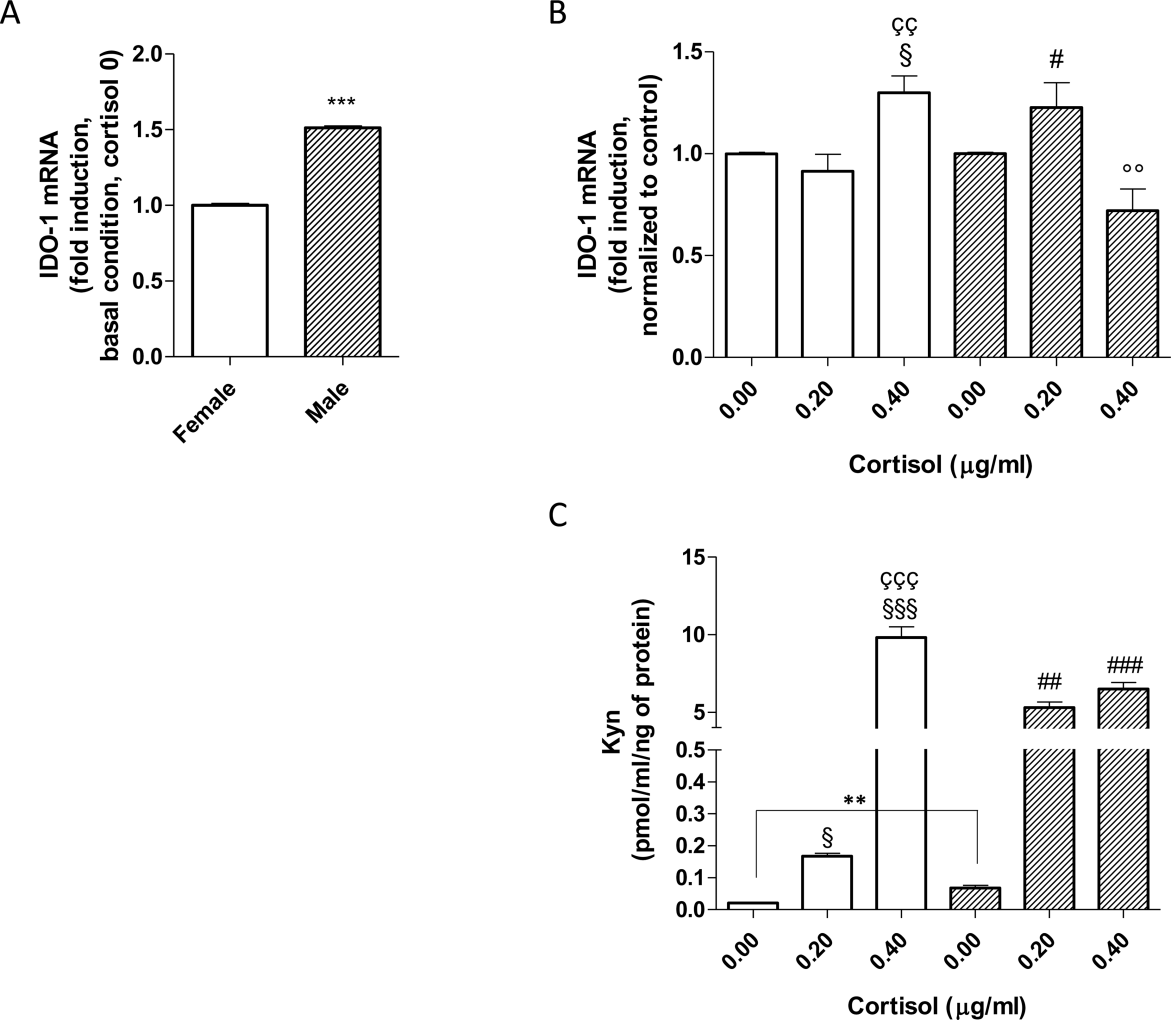
IDO-1 mRNA expression and kynurenine production following cortisol challenges. The white bars represent the mean ± SEM of three values performed in triplicate in female PBMCs; the striped bars represent the mean ± SEM of three values performed in triplicate in male PBMCs. *** p<0.001 or ** p<0.01 vs female cells (t-test); § p<0.05 vs non-stimulated female cells (One-Way Anova, Newman-Keuls post-test); # p<0.05 or ## p<0.01 or ### p<0.001 vs non-stimulated male cells (One-Way Anova, Newman-Keuls post-test); çç p<0.01 or ççç p<0.001 vs 0.2 microg/ml cortisol treated female cells (t-test); °° p<0.01 vs 0.2 microg/ml cortisol treated male cells (t-test). For IDO-1, Two-way ANOVA test (sex, treatment): interaction P value < 0.0001; F = 14.35; DFn = 2. For Kyn, Two-way ANOVA test (sex, treatment): interaction p value = 0.0005; F = 14.35; DFn = 2.

The relative IDO-1 mRNA expression in male and female PBMCs was performed by real-time RT-PCR analysis (Fig 4). In accordance with basal IL-6 levels, male PBMCs presented higher levels of IDO-1 with respect to female PBMCs (1.5-fold of the change vs female *** p<0,001) (Fig 4A). Physiological cortisol concentration did not affect the female IDO-1 mRNA expression; conversely, it was able to significantly increase the IDO-1 gene expression in male PBMCs (Fig 4B; p<0.05). Furthermore, the highest cortisol concentration produced a significant increase in the enzyme expression in female cells (p<0.01) and a significant decrease in male cells (p<0.01). Additionally, the two-way ANOVA test proved the different cortisol effects in male and female cells; the interaction was considered extremely significant (interaction P value < 0.0001; F = 14.35; DFn = 2). These results are in accordance with the pro-inflammatory secretion pattern shown by the male cells (Fig 3).

As IDO-1 catalyses the rate-limiting step of tryptophan oxidation yielding kynurenine, Kyn levels were assessed in the cell lysates. Kyn in the basal condition was significantly higher in male than in female PBMCs (**, p<0.01), completely in accordance with the results of IDO-1 mRNA basal expression. Furthermore, in accordance with IDO-1 gene expression, male and female PBMCs showed a significant increase in kynurenine levels in the presence of a physiological cortisol concentration (Fig 4). Notably, female PBMCs treated with the highest cortisol concentration released higher levels of kynurenine than male cells. Despite the decrease of IDO-1 gene expression, in male PBMCs, the highest cortisol concentration produced a significant release of Kyn. The two-way ANOVA test confirmed the differences between the two sexes; the interaction was considered extremely significant (interaction p value = 0.0005; F = 14.35; DFn = 2).

## Discussion

Men and women report different reactions to physical and mental stress [24]. Only few papers have described the sex influence on stress-mediated cytokine release in clinical studies and in *in vivo* models [25–31]. These data are difficult to compare, due to the intricate neurohumoral changes occurring in the response to acute stress conditions and to the difference in the experimental models and settings. In addition, the measurement of plasma cytokines is often not reliable since cytokine levels *in vivo* are frequently undetectable or highly variable. It has been reported that *in vitro* models provide more useful information about stimulus effects on specific cytokine production [32, 33]. In this light, the current study aimed to investigate the role of cortisol in stress-induced cytokine secretions using an *in vitro* cellular model. Previous research has been conducted in a similar PBMC model to investigate cytokine production in response to external agents [18, 34, 35]. Herein, pools of male and female PBMCs containing similar content of lymphocytes and monocytes were used as a valuable *in vitro* cellular model. In literature, the leukocyte count has been found similar between men and women, less then slightly higher proportion of eosinophils in men, and gender-related differences were not apparent for the monocyte cells [36, 37]. Furthermore, it has been reported that women had shown a slightly higher ratio of CD4 + to CD8 + cells (helper-suppressor ratio) than men. Interestingly, a low CD4/CD8 ratio is an immune risk phenotype [38–41]. However, a wide heterogeneity exists because not only sex, but also age, ethnicity, genetics, exposures, and infections may all affect the ratio [42–45].

The basal levels of cytokine production by PBMCs in our model were in accordance with the results in the literature. Such a model could allow for an accurate investigation of the stress-related modulation in cytokine secretion associated with sex. Interestingly, to the best of our knowledge, this is the first study that used a defined *in vitro* healthy PBMC model to assess the sex differences in cytokine secretion profiles following cortisol exposure. The sex-related differences in the inflammatory responses are widely documented in humans [46]; sex hormones affect innate immune cells modulating immune responses during infections [46–48]. Interestingly, genetic factors have been proposed in the orchestration of cytokine secretions. Indeed, some inflammation-related genes are expressed on sex chromosomes [49–51]. In addition, quantitative and qualitative differences in the cytokine release have been found in male and female children, suggesting that sex hormones are not the only factor responsible for sex differences [52]. In this light, a purpose of the present study was to investigate the cellular response excluding divergent and fluctuating sex hormone levels to focus the attention on specific genetic sex-related differences.

Cytokines may have different pro- or anti-inflammatory effects [53]. For instance, IL-6 is one of the major pro-inflammatory cytokines released during the first minutes after an injury, and it has been widely accepted as a marker of acute immune system activation [54]; IL-10 is an anti-inflammatory cytokine with pleiotropic effects in immunoregulation and inflammation [55]. Our data evidenced that following the cortisol challenge, male cells showed a secretion profile more directed to pro-inflammatory cytokines; indeed, an increase in IL-6 and IL-8 cytokine secretion was evidenced. In contrast, female PBMCs displayed a more pronounced anti-inflammatory secretion pattern, in fact in the presence of increasing cortisol concentrations a consistent IL-4 and IL-10 release was evidenced. These data are in line with the observations of Wolf and colleagues [56] reporting a higher stress response in males than in females. In addition, in our cellular model, male PBMCs generally secreted more cytokines with respect to female PBMCs; this is consistent with several previous *in vitro*, *ex vivo* and *in vivo* studies, demonstrating the production of greater levels of cytokines by male cells [57–65].

Actually, the cytokine secretion profile is regulated by complex control machinery to maintain a proper balance among inflammatory mediators. For instance, IL-10 has been previously demonstrated to inhibit the production of pro-inflammatory cytokines, such as IL-6 [66–67]. Additionally, the capacity to downregulate the production of IL-8 has been demonstrated for IL-4 and IL-10 in human PBMCs [68]. These findings may explain the cytokine levels found in our models; the cortisol treatments were able to increase the IL-4 and IL-10 secretion in female PBMCs, and these cytokines could in turn maintain lower levels of IL-6 and IL-8. In contrast, in male cells, the anti-inflammatory cytokines did not increase in response to cortisol, and thus they could not downregulate the pro-inflammatory cytokines.

Concerning the kynurenine pathway, inflammatory cytokines secreted during an immune response are able to increase the expression of IDO-1 that, in turn, creates an immunosuppressive microenvironment producing kynurenine [21,22]. Thus, IDO-1 has been generally considered an important counter-regulatory enzyme, which controls disproportionate immune responses [23]. The link between IDO-1 and cytokines is very complex; for example, IL-6 controls both the synthesis and degradation of IDO-1 [69]. In this light, the relation between inflammatory response and IDO-1 activity is mediated by multiple factors, especially when the system is perturbed with an external treatment or in pathological conditions. Indeed, at the baseline condition, in which the system is not perturbed by cortisol treatment, the higher level of IL-6 found in male cells corresponds to the higher level of IDO1 and Kyn (Fig 4).

Our data are in agreement with a previous study demonstrating that high concentrations of hydrocortisone and the cortisol analogous dexamethasone induced IDO-1 in human astrocytic cells [70]. In our model, a physiological cortisol concentration significantly increased the IDO-1 gene expression in male PBMCs; the highest cortisol concentration produced a significant increase in IDO-1 in female PBMCs but a significant decrease in male cells. Accordingly, with the male data, it has been recently demonstrated that a decrease in IDO-1 activity causes the overall production of pro-inflammatory cytokines, with a concomitant decrease in IL-10 [23].

Finally, female PBMCs treated with the highest cortisol concentration presented a kynurenine level almost double than that obtained in male cells. This finding may be of interest as the increased synthesis of detrimental tryptophan catabolites, such as kynurenine, may contribute to the onset of depression [71]. Indeed, it is well known that depression, stress and sex are strictly correlated; depression may be both a cause and an effect of psychological stress, and the occurrence of depression is more common in women than in men [72].

In conclusion, these results demonstrated the ability of cortisol to modulate differently the cytokine release pattern of male and female healthy PBMCs. In particular, the highest cortisol concentration affects male and female cells differently, shifting the cytokine release in favour of pro-inflammatory and anti-inflammatory molecule secretion profiles, respectively. Nevertheless such interesting results, some limitations of the study should be considered; for example, these findings could not be directly translate to clinic because isolated PBMC could respond differently from the whole blood. Furthermore, these data could be implemented by the cell model supplementation of other factors involved in stress conditions. Indeed, in humans, stress causes the activation of both the HPA axis and the sympathetic nervous system, resulting in the increase of also other neuroendocrine hormones, such as ACTH, CRH, somatostatin, catecholamines, and endogenous opioids that could in turn influence the cytokine release [73]. Moreover, in the complex *in vivo* response to stressors, the secreted cortisol concentrations depend on sex; recently, a study investigating young adults exposed to mental arithmetic plus public speaking stress has evidenced that women had lower amount of salivary cortisol levels than men [74]. Although additional studies investigating the sex-specific effects of neuroendocrine hormones in high stress will be needed, the current study provides useful information on the effects of sex on cytokine release induced by cortisol.
